# (FeNiMnMgCuCo)_3_O_4_ High-Entropy Cathode for Zinc-Ion Batteries

**DOI:** 10.3390/ma19081520

**Published:** 2026-04-10

**Authors:** Ningning Dong, Huanhuan Cui, Yuncheng Cai, Renzhi Jiang

**Affiliations:** Analytical & Testing Center, Huazhong University of Science and Technology, Wuhan 430074, China; lymj403@163.com (N.D.); choi_yc@163.com (Y.C.)

**Keywords:** high-entropy oxides, cathode, aqueous zinc-ion battery, electrochemical performance

## Abstract

**Highlights:**

**What are the main findings?**

- A novel high-entropy cathode (FeNiMnMgCuCo)_3_O_4_ is designed and synthesized for aqueous zinc-ion batteries.
- The material exhibits a high reversible capacity of 341.3 mA h g^−1^ at 0.1 A g^−1^.
- The material possesses excellent cycling stability (76.1% retention after 1000 cycles at 3 A g^−1^).
- Multi-element coexistence in mixed valence states and high configurational entropy (~1.78 R) enhance stability.
- The high-entropy design effectively promotes Zn^2+^ diffusion and redox kinetics while suppressing structural degradation during cycling.

**What are the implications of the main findings?**

- This work demonstrates a high-entropy cathode material with practical potential.
- This work provides new research insights for optimizing zinc-ion storage performance through composition design and entropy regulation.
- This work will be of significant interest to researchers in the fields of electrochemistry and energy storage.

**Abstract:**

As a result of the high safety, low cost, and environmental benignity, aqueous zinc-ion batteries are regarded as one of the most promising candidates for next-generation large-scale energy storage systems. However, their further development is constrained by performance bottlenecks in existing cathode materials, including capacity, cycle life, and reaction kinetics. In this study, a high-entropy design strategy is employed to synthesize the metal oxide (FeNiMnMgCuCo)_3_O_4_ with a cubic spinel structure, and its electrochemical performance as a cathode for zinc-ion batteries is systematically evaluated. The prepared (FeNiMnMgCuCo)_3_O_4_ high-entropy cathode exhibits high reversible capacity (341.3 mA h g^−1^ at 0.1 A g^−1^) and remarkable long-term cycling stability (76.1% retention after 1000 cycles at 3 A g^−1^). This work not only demonstrates a high-entropy cathode material with practical potential but also provides new research insights for optimizing zinc-ion storage performance through composition design and entropy regulation.

## 1. Introduction

To meet the urgent demand for sustainable energy storage technologies, developing novel electrochemical energy storage systems that combine high performance, high safety, and low cost is of great significance [1,2,3,4]. Although lithium-ion batteries have been extensively applied in consumer electronics and electric vehicles, their further advancement is hindered by multiple bottlenecks, including constraints on lithium resource scarcity, escalating manufacturing costs, and safety risks caused by flammable electrolytes [5,6,7,8,9]. In this context, aqueous zinc-ion batteries have been used as a promising alternative for next-generation energy storage, owing to their inherent safety, environmental benignity, cost-effectiveness, and abundant zinc resources [10,11,12,13,14,15,16]. However, the commercialization of zinc-ion batteries remains critically impeded by the poor performance of cathode materials. Conventional cathode materials (such as manganese-based and vanadium-based oxides) generally suffer from low intrinsic conductivity, high zinc ion (Zn^2+^) diffusion barriers, and irreversible structural degradation during cycling [17,18,19,20,21,22]. These issues make it difficult for the batteries to meet practical requirements in terms of actual capacity, rate performance, and long-term cycling stability [23]. Therefore, developing novel high-performance cathode materials capable of efficiently and reversibly storing zinc ions is crucial for advancing this technology toward practical application.

Over the past few years, the design concept of high-entropy materials has offered a promising strategy to breaking through the performance bottlenecks of traditional cathode materials. High-entropy oxides (HEOs) are composed of multiple primary metal ions in a near-equimolar ratio. Their distinctive “high-entropy effect” can induce significant lattice distortion and synergistic “cocktail effects”, thereby effectively modulating the electronic structure of the material and enhancing intrinsic electronic conductivity [21,24,25]. Simultaneously, the distorted lattice can effectively facilitate rapid ion transport, significantly improving the diffusion kinetics of Zn^2+^ [26]. More importantly, the high-entropy system has excellent configurational entropy stability thermodynamically, which can maintain the integrity of the structural framework during repeated ion insertion/extraction processes, thereby ensuring outstanding cycle life [27]. Collectively, these characteristics make high-entropy oxides a highly promising material platform for advanced energy storage applications.

Herein, we report the rational design and successful synthesis of a high-entropy spinel oxide (FeNiMnMgCuCo)_3_O_4_, and for the first time comprehensively evaluate its performance as a cathode of aqueous zinc-ion batteries. The precise chemical composition was determined via inductively coupled plasma optical emission spectroscopy (ICP-OES). Subsequently, the crystal structure, microstructure, elemental distribution, and chemical states of the material were systematically characterized using X-ray diffraction (XRD), field-emission scanning/transmission electron microscopy (SEM/TEM), energy-dispersive X-ray spectroscopy (EDS), and X-ray photoelectron spectroscopy (XPS). The electrochemical evaluation results revealed that the (FeNiMnMgCuCo)_3_O_4_ cathode delivers a high reversible specific capacity of 341.3 mA h g^−1^ at a current density of 0.1 A g^−1^. Notably, it retains 76.1% of its initial capacity after 1000 cycles at 3 A g^−1^, underscoring its robust electrochemical performance. This work not only confirms the effectiveness of the multi-component high-entropy design strategy in boosting the performance of electrode materials for zinc-ion batteries, but also offers fresh insights for developing next-generation high-performance energy storage materials through entropy engineering.

## 2. Materials and Methods

(FeNiMnMgCuCo)_3_O_4_ was synthesized via a hydrothermal method followed by subsequent heat treatment. In particular, stoichiometric amounts (1 mmol each) of MgCl_2_·6H_2_O, CoCl_2_·6H_2_O, MnCl_2_·4H_2_O, FeCl_3_·6H_2_O, NiCl_2_·6H_2_O, and CuCl_2_·2H_2_O were dissolved in 40 mL of deionized water. While stirring continuously, 1.25 mmol of hexadecyltrimethylammonium bromide (CTAB) was added as a surfactant, followed by the addition of 36 mmol of urea as a precipitating agent, forming a homogeneous precursor solution. The mixture was then transferred to a polytetrafluoroethylene (PTFE)-lined stainless steel autoclave, and subjected to a hydrothermal reaction at 135 °C for 6 h. After cooling naturally to room temperature, the precipitate was collected by vacuum filtration following repeated centrifugation and washing with alternating deionized water and ethanol. The resulting filter cake was dried in a vacuum oven at 60 °C for 24 h to obtain the precursor powder. Finally, the precursor was calcined in a muffle furnace under an air atmosphere, heated at a rate of 5 °C/min to 900 °C, and held at this temperature for 2 h to ensure annealing and crystallization, yielding the target high-entropy oxide powder.

The as-synthesized samples were characterized using various analytical techniques. The elemental composition was quantified using inductively coupled plasma optical emission spectrometry (ICP-OES, Prodigy Plus, Teledyne Leeman Labs, Windsor, CT, USA). The crystal structure was analyzed via X-ray diffraction (XRD, Empyrean, Malvern Panalytical, Eindhoven, The Netherlands) equipped with Cu Kα radiation (λ = 1.5406 Å) at a scanning rate of 5° min^−1^. The morphology and microstructure were examined using field-emission scanning electron microscopy (SEM, JSM-IT800, JEOL Ltd., Akishima, Japan) and transmission electron microscopy (TEM, ARM-200F, JEOL Ltd., Akishima, Japan). The elemental uniformity of the material was verified by energy-dispersive X-ray spectroscopy (EDS, Octane Elite Super, EDAX Inc., Mahwah, NJ, USA). The surface chemical states were investigated using X-ray photoelectron spectroscopy (XPS, AXIS-ULTRA DLD-600W, Shimadzu Corporation, Manchester, UK) with Al Kα excitation, with binding energies calibrated against the C 1s peak at 284.8 eV.

The electrochemical performance of the as-prepared samples was evaluated in CR2032 coin cells configured with the prepared material as the working electrode. Specifically, the obtained (FeNiMnMgCuCo)_3_O_4_ powder, carbon black, and polyvinylidene difluoride (PVDF) were mixed in an 8:1:1 weight ratio and thoroughly ground to form a homogeneous mixture. Subsequently, N-methyl-2-pyrrolidone (NMP) solvent was added to the mixture to create a homogeneous slurry. After that, the resulting slurry was spin-coated onto carbon paper, followed by drying in a vacuum oven at 75 °C for 14 h to fabricate the working electrode (with the prepared samples loading of 2.0–2.5 mg cm^−2^). Finally, CR2032 coin cells were assembled in ambient air using the prepared working electrode, a glass fiber separator, an aqueous electrolyte consisting of 2.0 M ZnSO_4_ and 0.1 M MnSO_4_, and a Zn foil as the counter electrode. The electrochemical tests and long-term cycling tests were conducted using a CHI600E electrochemical workstation (CH Instruments, Inc., Austin, TX, USA) and a LANHE CT2001A battery test system (LANHE Tech., Wuhan, China), respectively.

## 3. Results and Discussion

### 3.1. Material Characterization

The elemental composition of the as-synthesized (FeNiMnMgCuCo)_3_O_4_ powder was quantitatively analyzed via ICP-OES. As summarized in Table 1, the measured mass fractions of each metallic element are Fe (18.6%), Ni (11.9%), Mn (15.1%), Mg (5.7%), Cu (12.2%), and Co (16.1%). The corresponding molar fractions at cation sites are calculated to be Fe (0.22), Ni (0.14), Mn (0.18), Mg (0.15), Cu (0.13), and Co (0.18), which confirms the successful incorporation of all six target elements into the oxide lattice. Based on these atomic fractions, the configuration entropy (Δ*S_config_*) can be calculated using the Boltzmann formula: $\Delta S_{\mathrm{config}}=-R\sum_{i=1}^{n}x_{i}lnx_{i}$. Here, *R* represents the gas constant (8.314 J mol^−1^ K^−1^), and $x_{i}$ denotes the mole fraction of the *i*-th element. The calculated configurational entropy value is approximately 1.78 R, which is in line with the thermodynamic definition of a high-entropy material (typically considered high-entropy when configuration entropy exceeds 1.5 R). Therefore, it can be concluded that the material has a high entropy characteristic.

**Figure 1:**
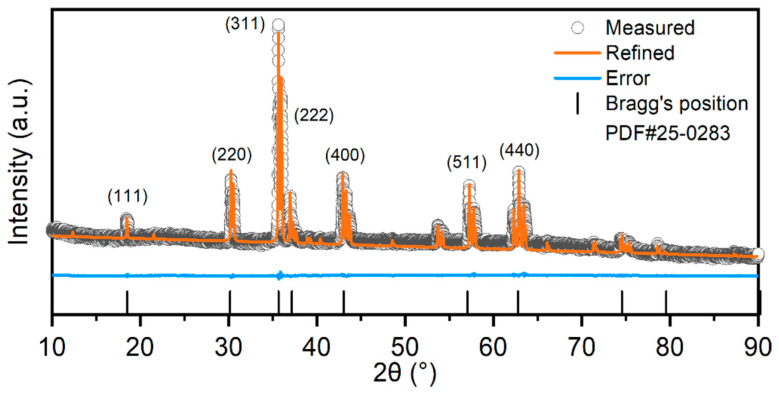
XRD pattern of (FeNiMnMgCuCo)_3_O_4_.

The crystal structure of the as-synthesized samples was investigated via X-ray diffraction (XRD). As illustrated in Figure 1, the XRD pattern exhibits sharp and intense diffraction peaks at 18.2°, 30.2°, 35.3°, 36.9°, 42.9°, 57.2°, and 62.8°, indicating good crystallinity of the material. All observed diffraction peaks corresponded accurately with the standard pattern for the cubic spinel structure (PDF#25-0283, CuFe_2_O_4_), and are indexed to the (111), (220), (311), (222), (400), (422), (511), and (440) crystal planes, respectively. Notably, no discernible diffraction signals from other impurities were detected, suggesting that the synthesized (FeNiMnMgCuCo)_3_O_4_ exhibits a single cubic spinel phase with high phase purity.

**Figure 2:**
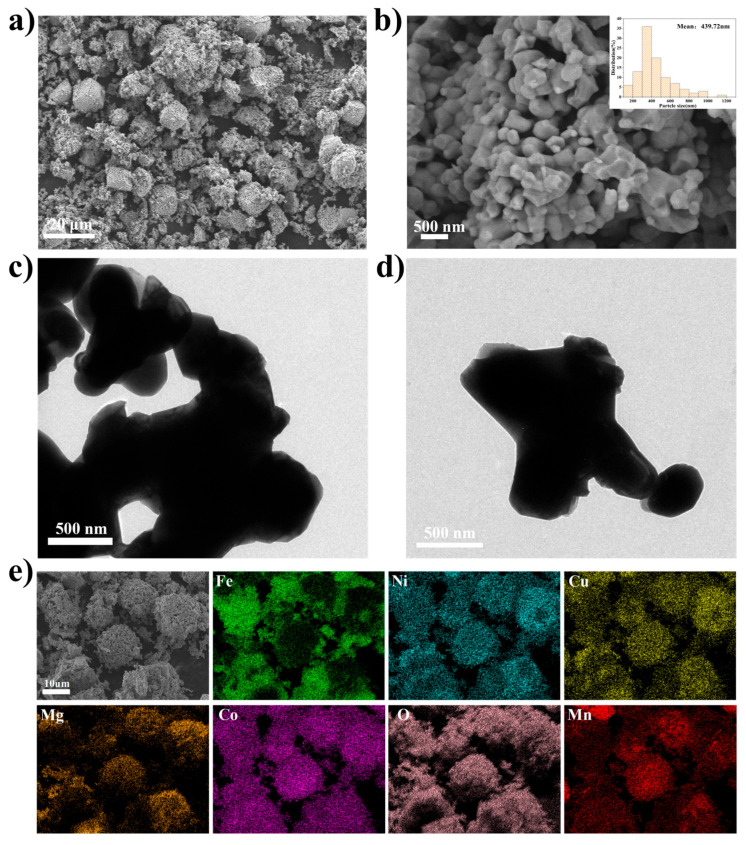
(**a**,**b**) SEM images, (**c**,**d**) TEM images, (**e**) SEM images and corresponding EDS mappings of (FeNiMnMgCuCo)_3_O_4_.

The morphology and microstructure of the as-synthesized samples were further investigated by field-emission scanning electron microscopy (SEM) and field-emission transmission electron microscopy (TEM). The low-magnification SEM image (Figure 2a) reveals that the sample consists of irregular agglomerates composed of numerous nanoparticles. The high-magnification SEM image (Figure 2b), along with the corresponding particle size distribution histogram (inset), demonstrates that the constituent particles are solid nanoparticles with good crystallinity, and their diameters are mainly in the range of 200 to 700 nm. The TEM images (Figure 2c,d) corroborate these observations. Collectively, these results indicate that (FeNiMnMgCuCo)_3_O_4_ exhibits a microstructure composed of densely packed, crystalline nanoparticles. This nanoscale particle structure enhances the interfacial area between the electrode and electrolyte, thereby facilitating efficient ion and electron transport. Furthermore, the elemental uniformity of the material was verified by energy-dispersive spectroscopy (EDS). The result (Figure 2e) shows that the six elements are evenly distributed within the representative particles, verifying that all six cations are homogeneously incorporated into the (FeNiMnMgCuCo)_3_O_4_ lattice.

**Figure 3:**
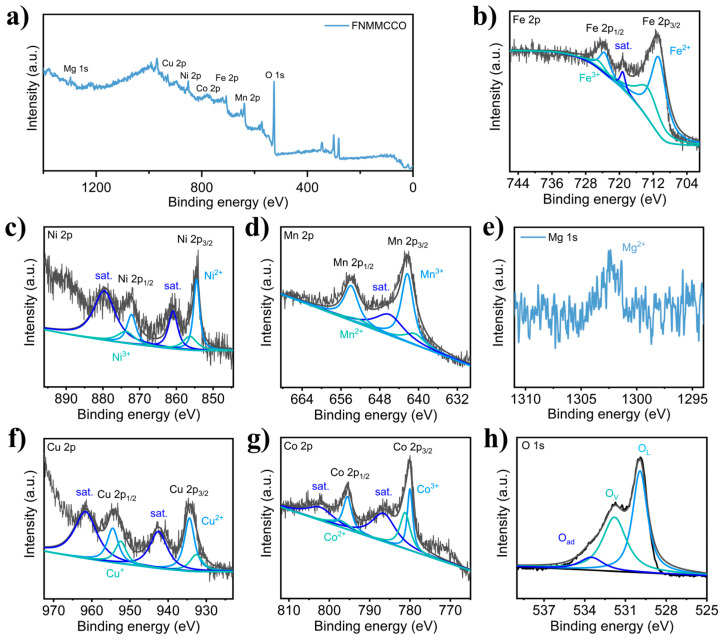
(**a**) XPS survey spectrum of (FeNiMnMgCuCo)_3_O_4_. XPS spectra of (**b**) Fe 2p, (**c**) Ni 2p, (**d**) Mn 2p, (**e**) Mg 1s, (**f**) Cu 2p, (**g**) Co 2p, and (**h**) O 1s.

X-ray photoelectron spectroscopy (XPS) was utilized to systematically investigate the elemental composition and surface chemical valence states of (FeNiMnMgCuCo)_3_O_4_. As shown in Figure 3a, the survey spectrum displays the presence of characteristic peaks for Fe, Ni, Mn, Mg, Cu, and Co, confirming the coexistence of all six target metal elements, and aligning with the results of EDS analysis. In the high-resolution Fe 2p spectrum (Figure 3b), the binding energy peaks observed at 710.9 eV and 723.7 eV are assigned to the 2p3/2 and 2p1/2 orbitals of Fe^2+^, respectively. Concurrently, the peaks at 714.2 eV and 725.6 eV are attributed to the Fe^3+^ state, while a distinct satellite peak at 719.1 eV further corroborates the coexistence of mixed Fe valence states.

The Ni 2p spectrum (Figure 3c) exhibits characteristic Ni^2+^ peaks at 854.5 eV (Ni 2p3/2) and 872.1 eV (Ni 2p1/2), while contributions from Ni^3+^ are observed at 856.3 eV and 873.9 eV, accompanied by satellite peaks at 860.8 eV and 879.7 eV, respectively. Similarly, the Mn 2p spectrum (Figure 3d) can be decomposed into two pairs of peaks. The peaks appearing at 640.9 eV and 652.6 eV are attributed to Mn^2+^, while the peaks at 642.2 eV and 653.9 eV belong to Mn^3+^, with their satellite peaks located at 646.3 eV.

The Mg 1s spectrum (Figure 3e) displays a symmetrical peak at 1302.3 eV, which is consistent with the Mg^2+^ state. Notably, the relative signal intensity of Mg observed via XPS is lower than expected based on the stoichiometry determined by inductively coupled plasma optical emission spectroscopy (ICP-OES). This discrepancy can be attributed to the following reasons. First, the sensitivity of XPS is dictated by the photoionization cross-section of specific orbitals. Since Mg possesses a significantly lower atomic number compared to the co-existing transition metals, the photoionization cross-section of the Mg 1s orbitals is substantially smaller than that of the Fe, Ni, and Co 2p orbitals. Consequently, the probability of photoelectron emission from Mg atoms is inherently lower, resulting in weaker peak intensities even at comparable concentrations. Second, this effect is exacerbated by matrix effects and signal competition. The dominant signals from transition metals can overshadow the relatively weak Mg response. Furthermore, the Mg region is susceptible to high background noise from secondary electrons and potential interference from surface contaminants. Collectively, these factors result in an apparent underestimation of Mg content in XPS relative to bulk techniques like ICP-OES.

The analysis of the Cu 2p spectrum (Figure 3f) indicates that the stronger peaks at 934.3 eV and 954.5 eV are typical characteristics of Cu^2+^, along with the prominent satellite peaks (located at 942.6 eV and 961.3 eV). As for the peaks located at 932.6 eV (Cu 2p3/2) and 952.5 eV (Cu 2p1/2), it is difficult to determine whether they belong to Cu^+^ or Cu^0^ solely based on the binding energy. Combined with the preparation conditions of the material (high-temperature calcination under air conditions), it can be inferred that these peaks correspond to Cu^+^. The peaks at 779.9 eV (Co 2p3/2) and 795.5 eV (Co 2p1/2) in the Co 2p spectrum (Figure 3g) indicate the presence of Co^3+^, while the peaks at 781.2 eV and 797.2 eV correspond to Co^2+^, with their satellite peaks located at 786.3 eV and 801.8 eV respectively.

Deconvolution analysis of the O 1s spectrum (Figure 3h) further reveals the diversity of the oxygen environment. The main peak at 529.9 eV corresponds to lattice oxygen (OL), the peak at 531.8 eV is attributed to oxygen vacancies (Ovs), while the weak peak at 533.9 eV relates to surface-adsorbed oxygen (Oad). The presence of oxygen vacancies typically enhances electronic conductivity and facilitates ion migration, which is beneficial for electrochemical performance. Collectively, the XPS test results indicate the presence of all the expected metal elements, and Fe, Ni, Mn, Cu, and Co coexist in mixed valence states. This multi-valence environment, combined with a significant oxygen vacancy concentration, jointly constitutes the unique electronic structure of the high-entropy oxide. It is expected to facilitate the enhancement in the intrinsic electrical conductivity of the material and optimize its electrochemical reaction activity.

### 3.2. Electrochemical Performance

**Figure 4:**
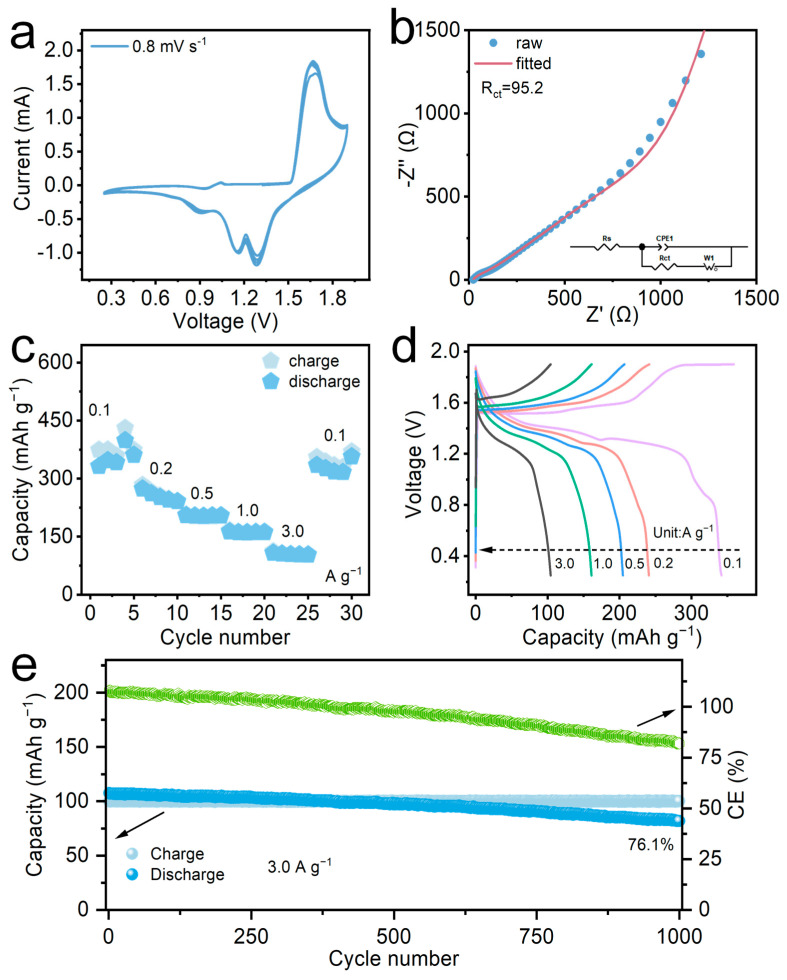
(**a**) CV curve. (**b**) EIS curve. (**c**) Rate capability. (**d**) Charge–discharge curves. (**e**) Long cycle performance.

The electrochemical performance of the (FeNiMnMgCuCo)_3_O_4_ high-entropy cathode was systematically evaluated by using CR2032 coin cells, which were assembled with (FeNiMnMgCuCo)_3_O_4_ as the cathode, zinc foil as the anode, and a 2.0 M ZnSO_4_ + 0.1 M MnSO_4_ aqueous solution as the electrolyte. In zinc-ion batteries, the pre-addition of Mn^2+^ to the electrolyte can effectively suppresses the dissolution and loss of manganese in the cathode material through the common-ion effect, thereby significantly enhancing the cycling stability. The Zn^2+^ storage behavior was comprehensively investigated through cyclic voltammetry (CV), electrochemical impedance spectroscopy (EIS), galvanostatic charge–discharge (GCD), and long-cycle stability tests. The CV curve (Figure 4a) displays a pair of distinct and stable redox peaks at approximately 1.66/1.28 V, corresponding to the reversible extraction/insertion process of Zn^2+^. This redox potential range aligns closely with the reaction characteristics of typical manganese-based oxides in zinc sulfate electrolytes. Combined with the fact that multiple elements (Fe, Ni, Co, Cu) in XPS exhibit mixed valence states, it can be inferred that the oxidation–reduction peaks may correspond to a complex multi-electron reaction centered on Mn and facilitated by the synergy of other transition metal elements. The EIS test results (Figure 4b) reveal a small semicircle in the high-frequency region, corresponding to a low charge transfer resistance, indicating rapid charge transfer kinetics at the electrode–electrolyte interface. After equivalent circuit fitting, the charge transfer resistance (R_ct_) is determined to be 95.2 Ω, further confirming the rapid interface charge transfer kinetics. The rate performance curves shown in Figure 4c further imply the favorable kinetic properties of this material. The (FeNiMnMgCuCo)_3_O_4_ electrode exhibits reversible capacity response over a wide current density range, highlighting its excellent rate performance. At current densities of 0.1, 0.2, 0.5, 1.0, and 3.0 A g^−1^, the specific discharge capacities reached 341.3, 240.7, 204.5, 160.8, and 104.1 mA h g^−1^, respectively (Figure 4d). Long-cycle performance testing further corroborates the structural stability of this electrode. As illustrated in Figure 4e, after 1000 cycles at a high current density of 3.0 A g^−1^, the electrode retains a discharge specific capacity of 81.9 mA h g^−1^, corresponding to a high-capacity retention rate of 76.1%, demonstrating excellent long-cycle life and structural robustness. As for the continuous decay in Coulombic efficiency (CE) observed during cycling, it might be attributed to the cumulative effects of transition metal dissolution (particularly Mn and Fe via disproportionation reactions), parasitic hydrogen evolution reactions (HERs), and the instability of the solid–electrolyte interphase (SEI) in aqueous systems. Nevertheless, compared with the cathodes recently reported for ZIBs, (FeNiMnMgCuCo)_3_O_4_ has a high specific capacity and a wide voltage range (Table 2). The overall electrochemical performance indicates that the (FeNiMnMgCuCo)_3_O_4_ high-entropy oxide cathode successfully balances high capacity, rapid reaction kinetics, and long-term cycling stability. These can be attributed to the stable crystal framework inherent to its high-entropy structure, optimized electronic conductivity, and a multi-ion-coordinated redox reaction mechanism, which collectively mitigate structural degradation and capacity loss during the cycling process, underscoring the significant application potential of this material system in aqueous zinc-ion batteries.

## 4. Conclusions

In summary, a high-entropy spinel oxide, (FeNiMnMgCuCo)_3_O_4_, has been rationally designed, successfully synthesized, and systematically evaluated as a promising cathode material for aqueous zinc-ion batteries. Comprehensive characterization confirms that the material exhibits a single cubic spinel phase with irregular nanoparticle morphology, along with a homogeneous distribution of all six constituent metal elements (Fe, Ni, Mn, Mg, Cu, Co) in mixed-valence states. The high configurational entropy (~1.78 R) provides a thermodynamic foundation for structural stability. The (FeNiMnMgCuCo)_3_O_4_ cathode delivers a high reversible capacity of 341.3 mA h g^−1^ at 0.1 A g^−1^, while retaining 76.1% of its initial capacity after 1000 cycles at a high current density of 3.0 A g^−1^, underscoring its exceptional long-term cycling stability. This study not only validates the effectiveness of the multi-component high-entropy strategy in enhancing zinc-ion battery cathode material performance but also provides new insights for designing high-performance, long-life energy storage materials through entropy engineering.

## Figures and Tables

**Table 1:** ICP-OES results of (FeNiMnMgCuCo)_3_O_4_.

Elements	Normalized Molar Fraction	Normalized Atom	Wt. (%)
Fe	0.22	1.32	18.6
Ni	0.14	0.81	11.9
Mn	0.18	1.09	15.1
Mg	0.15	0.92	5.7
Cu	0.13	0.76	12.2
Co	0.18	1.09	16.1

**Table 2:** Performance comparison of recently reported cathodes for ZIBs with (FeNiMnMgCuCo)_3_O_4_.

Cathode	Electrolyte	Voltage Window (V vs. Zn^2+^/Zn)	Cycles	Capacity (mA h g^−1^)	Ref.
δ-MnO_2_	1 M Zn(TFSI)_2_ + 0.1 M Mn(TFSI)_2_	1.0–1.8	93% capacity retention at 6.16 A g^−1^ after 4000 cycles	171 mA h g^−1^ at 1.54 A g^−1^	[28]
α-MnO_2_	3 M ZnSO_4_	0.2–2.2	80.1% capacity retention at 1 A g^−1^ after 6000 cycles	323 mA h g^−1^ at 0.1 A g^−1^	[29]
commercial V_2_O_5_	3 M Zn(CF_3_SO_3_)_2_	0.2–1.6	91.1% capacity retention at 5 A g^−1^ after 4000 cycles	465 mA h g^−1^ at 0.2 A g^−1^	[30]
Prussian blue analog (HEPBA)	2 M ZnSO_4_ + 0.2 M MnSO_4_	0.5–1.8	84.7% capacity retention at 0.5 A g^−1^ after 600 cycles	132.1 mA h g^−1^ at 0.1 A g^−1^	[31]
(FeNiMnMgCuCo)_3_O_4_	2 M ZnSO_4_ + 0.1 M MnSO_4_	0.25–1.9	76.1% capacity retention at 3 A g^−1^ after 1000 cycles	341.3 mA h g^−1^ at 0.1 A g^−1^	This work

## Data Availability

The original contributions presented in this study are included in the article. Further inquiries can be directed to the corresponding author.
